# Gut microbiota resilience and recovery after anticancer chemotherapy

**DOI:** 10.20517/mrr.2022.23

**Published:** 2023-05-06

**Authors:** Sara Roggiani, Mariachiara Mengoli, Gabriele Conti, Marco Fabbrini, Patrizia Brigidi, Monica Barone, Federica D'Amico, Silvia Turroni

**Affiliations:** ^1^Microbiomics Unit, Department of Medical and Surgical Sciences, University of Bologna, Bologna 40138, Italy.; ^2^Unit of Microbiome Science and Biotechnology, Department of Pharmacy and Biotechnology, University of Bologna, Bologna 40126, Italy.

**Keywords:** Gut microbiota, chemotherapy, cancer, recovery, resilience, probiotics, prebiotics, fecal microbiota transplantation

## Abstract

Although research on the role of the gut microbiota (GM) in human health has sharply increased in recent years, what a “healthy” gut microbiota is and how it responds to major stressors is still difficult to establish. In particular, anticancer chemotherapy is known to have a drastic impact on the microbiota structure, potentially hampering its recovery with serious long-term consequences for patients’ health. However, the distinguishing features of gut microbiota recovery and non-recovery processes are not yet known. In this narrative review, we first investigated how gut microbiota layouts are affected by anticancer chemotherapy and identified potential gut microbial recovery signatures. Then, we discussed microbiome-based intervention strategies aimed at promoting resilience, *i.e.*, the rapid and complete recovery of a healthy gut microbial network associated with a better prognosis after such high-impact pharmacological treatments.

## INTRODUCTION

It is now a fact that the human gut microbiota (GM), *i.e.*, the over trillion microbial cells, mainly bacteria, together with archaea, fungi, and viruses that are hosted in the gastrointestinal tract, plays a multifactorial role in our physiology, from regulation of metabolism to the education and modulation of the immune system, and not least of the nervous system, just to name a few^[1,2]^. Despite this awareness and the significant advances in the compositional and functional profiling of GM, the precise features of a “healthy” (otherwise called eubiotic) GM are still far from being defined^[3]^. This is mainly due to the intrinsically plastic nature of GM, which allows it to respond to external perturbations (of limited duration and severity) by oscillating between different stable states associated with health^[4]^. Indeed, GM differs over time and between individuals, in relation to factors such as diet, lifestyle, environmental exposure, or more generally, what we could define as the exposome^[5]^. Nonetheless, studies on GM fluctuations in response to these drivers and in comparison with disease settings have made it possible to identify some characteristics shared by eubiotic GMs all over the world, namely a high level of diversity, high relative abundances of bacteria capable of producing beneficial metabolites (mainly short-chain fatty acids - SCFAs, bioactive small molecules with a pluripotent role in human physiology)^[6]^ and low proportions of overt or opportunistic pathogens^[7-12]^. These features are in fact associated with a healthy gut whose epithelial barrier is intact and whose immune system is adequately trained. In particular, diversity predominantly contributes to the stability of microbial communities, as it allows buffering invasions and facilitates efficient use of resources and, in general, a certain level of functional redundancy, thus supporting the ability to recover rapidly and fully from perturbations, i.e., resilience^[4]^.

However, under certain conditions, the factors listed above, depending on their duration and intensity, can seriously compromise the stability of the GM, pushing it towards an unstable state that, once the perturbation has ceased, can recover to its original state or stabilize in a new alternative, healthy or vice versa dysbiotic (or disease-associated) state^[4,13]^. More precisely, the GM response to perturbations can be of 3 types: resilience, resistance, or hysteresis. As anticipated above, resilience is the property of a microbial ecosystem that defines how quickly and to what extent it will recover its initial taxonomical and/or functional composition following perturbations^[14]^. The GM is defined as resistant when it remains substantially unchanged in the face of perturbations. Finally, when the GM fails to recover from disturbance-induced changes and reaches a new stable state, which can be healthy or unhealthy^[4]^, this is called hysteresis^[15]^. For example, the GM is generally resilient to acute travel-related perturbations [such as dietary changes, contamination of ingested food or water, and possible drug intake (e.g., malaria prophylaxis)], as upon returning home, it tends to recover its original state rather quickly^[15,16]^. Conversely, antibiotic exposure can dramatically perturb GM, in a manner strongly dependent on the initial state, with potentially long-lasting effects^[4,17,18]^. In particular, vancomycin use has been associated with a depletion of the relative abundance of beneficial butyrate-producing taxa, such as *Coprococcus eutactus* and *Faecalibacterium prausnitzii*, along with a decrease in plasma butyrate concentration, which persisted at 2-month follow-up^[18]^. Moreover, bacterial species such as *Bacteroides thetaiotaomicron* and *Bifidobacterium adolescentis* have recently been shown to be associated with ecological recovery after antibiotic therapy, as they were able to support (and boost) the repopulation of other gut species through specific carbohydrate-degradation and energy-production pathways^[19]^.

In addition to antibiotics, anticancer chemotherapy can be considered another major nuisance for GM. Chemotherapeutic agents can, in fact, have direct effects on the composition of GM as well as destroying gut homeostasis, compromising the integrity of the mucosal barriers and allowing the translocation of microorganisms into the lamina propria and potentially throughout the body, with induction of a strong inflammatory state^[20,21]^. Depending once again on the initial layout and the specific dynamics that are established during treatment, the GM can favor the therapeutic response or vice versa, the onset of adverse events, including death^[22-24]^. However, what exactly are the GM signatures associated with a favorable prognosis, a proper recovery process and the underlying driving forces are not yet fully understood. Understanding these aspects may provide valuable opportunities to rationally design microbiome-based intervention strategies aimed at strengthening GM resistance or promoting its ecological recovery, increasing the resilience of healthy states or overcoming that of unhealthy states.

**Figure 1:**
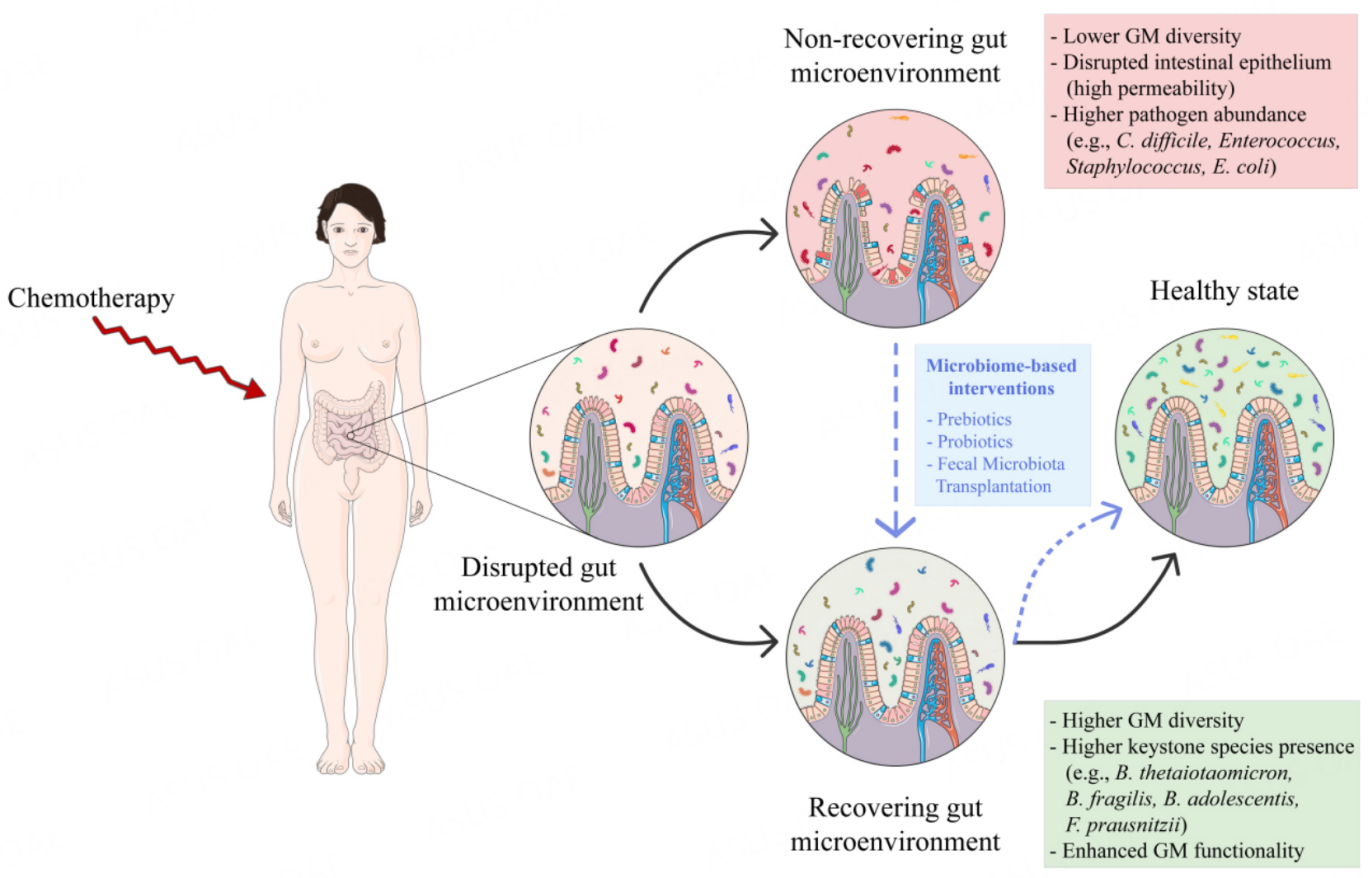
Gut microbiota recovery after chemotherapy treatment. High-impact pharmacological treatments such as chemotherapy can cause profound disturbance of the intestinal environment, including inflammation, breakdown of mucosal barriers, and shifts in the gut microbiota composition. Depending on the initial microbiota state and other treatment-related factors, this perturbation may lead to the establishment of a stable state of “recovery” or “non-recovery”. The recovery state is generally characterized by greater resilience due to greater microbiota diversity and the presence of founders or keystone taxa (e.g., *Bacteroides thetaiotaomicron*, *Bacteroides fragilis*, *Bifidobacterium adolescentis*, and *Faecalibacterium prausnitzii*), able to favor the repopulation of other commensals, for rapid restoration of a properly functioning eubiotic ecosystem. The non-recovery state is featured by dysbiotic traits such as lower gut microbiota diversity, increased proportions of pathobionts (e.g., *Clostridioides difficile*, *Enterococcus*, *Staphylococcus*, and *Escherichia coli*), whose colonization and expansion may be promoted by the loss of competing beneficial commensals in an inflammatory environment, and a disrupted intestinal epithelium. Microbiome-targeted interventional strategies (e.g., prebiotics, probiotics, and fecal microbiota transplantation) may facilitate the transition from a non-recovery to a recovery state, thus accelerating the re-establishment of a healthy gut microbiota layout and protecting against the long-term consequences of chemotherapy. The figure was partly generated using Servier Medical Art provided by Servier, licensed under a Creative Commons Attribution 3.0 unported license, and images from Flaticon resources. GM: gut microbiota.

In this narrative review, we focus on anticancer chemotherapy as one of the most profound and impactful ailments for GM. We discuss post-treatment recovery and non-recovery processes of GM in the context of different cancers, paying attention to the main factors influencing these dynamics. Finally, we summarize the current evidence on microbiome-based intervention strategies aimed at supporting rapid and full restocking of a eubiotic GM [Figure 1].

## CHEMOTHERAPY-INDUCED ALTERATIONS IN THE GM AND POTENTIAL SIGNATURES OF RECOVERY

In recent years, GM and its metabolites have received a *crescendo* of attention for their involvement in cancer initiation and progression, as well as in anticancer therapy outcomes^[25]^. In particular, to date, it is known that there is a bidirectional relationship between GM and anticancer chemotherapy, with the former influencing the efficacy of the treatments, by modulating the immune system and impacting drug pharmacokinetics, and the latter seriously undermining the microbiota stability with potentially long-term consequences for health^[26]^. Such chemotherapy-related effects on GM can be direct or indirect, *i.e.*, mediated through high-impact side effects, including gastrointestinal toxicity, malnutrition, myelosuppression, hepatoxicity, and neurological symptoms. Indeed, the most common side effects of chemotherapy are abdominal pain, abdominal bleeding, nausea, infections, and diarrhea which are experienced by nearly 80% of oncological patients during treatments. Underlying causes include destruction of the gastrointestinal tract mucosal barrier, with possible onset of mucositis (*i.e.*, inflammation of the mucosa), epithelial cell death, and malabsorption, which fuel (and are fuelled by) the disruption of the GM ecosystem^[27]^. In fact, chemotherapy acts as a strong stressor on the GM, pushing it towards an unstable and transient state, after which it may or may not recover its initial state (*i.e.*, show or not resilience). As anticipated above, chemotherapy-related perturbations can also lead to a new stable state of health or malaise (so-called hysteresis), which could further favor disease development. How the GM responds to chemotherapy is the result of a complex and multifactorial process that depends on many variables, not just those related to the chemotherapy regimen (*i.e.*, type and dosage of anticancer drugs), but also the type of cancer, the stage of the disease, the co-administration of other drugs, other patient data (demographic, anthropometric, biochemical, genetic, immunological, dietary, etc.), and, of course, the baseline GM configuration. In particular, patients undergoing chemotherapy usually have significantly reduced oral intake due to the side effects of such aggressive treatment, including those mentioned before, such as enteral mucositis and nausea^[28,29]^. This deterioration of patients’ nutritional status may exacerbate GM dysbiosis and mucosal injury caused by mucositis, ultimately worsening clinical outcomes^[30]^.

In this scenario, understanding GM fluctuations during anticancer treatments is critical to increase the resilience of healthy states (or surpass that of unhealthy states) for rapid and complete restoration of a eubiotic GM configuration associated with a better prognosis. However, also due to the recent awareness of the relationship between gut microbes and anticancer chemotherapy, relatively few studies are present in the literature (please, see Table 1 for a summary of the studies herein discussed). Nevertheless, chemotherapy undoubtedly leads to a reduction in the diversity of the GM^[23,24,31,32]^, with a consequent potential loss of functional redundancy (although mostly not experimentally verified), which appears crucial for the stability of the GM during perturbations and, therefore, for its resilience. The first studies in this field were conducted on murine models treated with different chemotherapeutics, showing increased levels of *Bacteroides*^[33-36]^. However, *Bacteroides* were also shown to decrease following chemotherapy treatments, along with some beneficial microbes such as *Bifidobacterium* and *Lactobacillus* spp.^[37-39]^. With regard to human studies, Zwielehner *et al.* analyzed the GM profile of 17 patients with different types of cancer before and after chemotherapy. Pharmacological treatment promoted a slight increase in *Bacteroides* spp., as well as pathobionts not detected before treatment (e.g., *Clostridioides difficile, Enterococcus faecium*)^[40]^. Fei *et al.* found decreased microbial richness (*i.e.*, the total number of species in a given sample) and diversity (which refers, depending on the metric used, to either richness or evenness, or both, and can also take into account phylogenetic relationships), in colorectal cancer patients receiving antimetabolites and platinum-based chemotherapeutic agents^[41]^, while post-treatment enrichment of *Bacteroides* was found^[42]^. An elegant study from Youssef *et al.*^[43]^ compared the GM profile of patients with untreated gastrointestinal malignancies (*i.e.*, cancer of the stomach, pancreas, small intestine, colon, and rectum) with that of patients treated with chemotherapy and/or radiotherapy and healthy controls. Treated individuals, compared to untreated ones, had a significantly higher relative abundance of potentially beneficial taxa belonging to the *Lactobacillaceae* family, such as *Lactobacillus*. One might be tempted to speculate that the chemotherapy regimen is beneficial to the GM; however, it is far more likely that this increase is due to pre-treatment GM status or other conditions that facilitated a prompt GM recovery. Additionally, chemotherapy-treated patients exhibited decreased levels of health-associated microbes, namely *Bifidobacterium*, *Ruminoclostridium*, *Lachnoclostridium,* and *Oscillobacter,* compared to healthy controls^[43]^. Partially conflicting results emerged in the study by Stringer *et al.*^[44]^, in which reduced proportions of *Lactobacillus* spp., *Bacteroides* spp., *Bifidobacterium* spp., and *Enterococcus* spp., and increased abundances of *Staphylococcus* spp. and *Escherichia coli* have been observed in patients undergoing chemotherapy for the treatment of several types of cancer. Platinum-based chemotherapy has also been shown to exert a detrimental effect on the GM of women with epithelial ovarian cancer, particularly with decreased proportions of health-promoting SCFA-producing taxa, such as *Lachnospiraceae* and *Ruminococcaeae*. Notably, an increased level of *Coriobacteriaceae* and *Bifidobacterium* over time was associated with platinum resistance and non-response to therapy^[23]^*.* Data on *Bifidobacterium*, a well-known probiotic genus, appear contradictory but could be related to its ability to produce lactate, a key oncometabolite in several cancers, and its anti-inflammatory role, and thus possibly a poor ability to promote antitumor immune responses. The results of D’Amico *et al.*^[23]^ were confirmed by Tong *et al.*^[45]^, who showed that the GM of ovarian cancer patients undergoing multiple cycles of chemotherapy was characterized by a higher relative abundance of *Collinsella*, belonging to the *Coriobacteriaceae* family, in addition to *Blautia* and *Bacteroides*, with the latter reported to be increased in other cancer types as well. It is also worth noting that oxaliplatin, a platinum-based drug, was found to be overall more aggressive than 5-fluorouracil, irinotecan, and calcium folinate, in terms of intestinal injury and GM dysbiosis^[46]^. Moreover, the GM dynamics were studied in adult patients with acute myeloid leukemia, treated with antimetabolites or hypomethylating agents, and subjected to antimicrobial prophylaxis^[47]^. Most frequently, opportunistic pathogens (e.g., *Staphylococcus*, *Enterobacter*, and *Escherichia*) have been found to make up over 30% of intestinal bacteria, but again an overabundance of *Lactobacillus* was observed, potentially related to recovery. However, current evidence on the ecological role of *Lactobacillus*, with particular regard to its resilience to stressors or its ability to promote GM recovery, is still inconclusive.

**Table 1:** Clinical studies investigating gut microbiota variations during chemotherapy treatments

**Study**	**Cancer type**	**Treatment**	**Number of patients**	**Main results**
D'Amico *et al.*^[23]^	Epithelial ovarian cancer	Surgery + chemotherapy with platinum and taxane compounds	24	- Treatment-related decrease in health-promoting SCFA-producing taxa, such as *Lachnospiraceae* and *Ruminococcaeae* - Increased levels of *Coriobacteriaceae* and *Bifidobacterium* over time were associated with platinum resistance and non-response to therapy
Peled *et al.*^[24]^	Hematological malignancies	Various intensities of conditioning regimens before HSCT	1362	- Reduction in bacterial diversity after treatment - Non-recovers showed an increased risk of infections, aGvHD, and relapse
Biagi *et al.*^[31]^	Hematological malignancies (pediatric patients)	Conditioning regimens based on busulfan before HSCT	10	- Only 10% of pre-existing species resisted after HSCT, with *Bacteroides* spp. being the most represented among the persistent ones - A decrease in the relative abundance of health-associated taxa, such as *Faecalibacterium* and *Ruminococcus*, after HSCT - Pre-HSCT samples of aGvHD patients showed a lower abundance of *Parabacteroides* and *Bacteroides*
Biagi *et al.*^[32]^	Hematological malignancies (pediatric patients)	Conditioning regimens (busulfan, cyclophosphamide/fludarabine, total body irradiation) before HSCT	36	- Reduced microbial diversity, lower *Blautia* content, and increase in *Fusobacterium* abundance were predictive gut microbiota signatures of subsequent aGvHD occurrence
Zwielehner *et al.*^[40]^	Various types of malignancies (e.g., urothelial carcinoma, multiple myeloma, non-Hodgkin lymphoma, ovarian fibroma, leukemia, small intestinal tumor, rectal tumor, colon tumor)	Chemotherapy (antimetabolites, alkylating agents, monoclonal antibodies, corticosteroids, plant alkaloids, platinum-containing compounds, radiation therapy, anthracyclines, cytotoxic topoisomerase I and II inhibitors)	17	- Decreased species richness after chemotherapy in comparison with healthy individuals - Increase in *Bacteroides* spp*.* during chemotherapy - Decreased abundances of *Bifidobacterium* and *Clostridium* clusters IV and XIVa after chemotherapy - *Enterococcus faecium* increased following chemotherapy - The occurrence of *Clostridioides difficile* in 3/17 subjects was associated with a decrease in the genera *Bifidobacterium*, *Lactobacillus*, *Veillonella,* and the species *Faecalibacterium prausnitzii*
Fei *et al.*^[41]^	Stage III colorectal cancer	Chemotherapy (capecitabine + oxaliplatin)	17	- Patients with CID, compared with those who did not experience CID, had lower bacterial richness along with increased Proteobacteria, Gammaproteobacteria, Enterobacteriales, and *Enterobacteriaceae* (particularly *Klebsiella pneumoniae*) - Patients who did not develop CID had increased abundances of Clostridia, Clostridiales, *Ruminococcaceae*, and Bacteroidetes - In general, an increased abundance of Bacteroidales, *Bacteroidaceae,* and *Bacteroides* was observed
Deng *et al.*^[42]^	CRC patients before any chemotherapy treatments, CRC patients surgically treated, and CRC patients treated with chemotherapy	Chemotherapy (5-FU + oxaliplatin)	17 (CRC before chemotherapy) 14 (CRC after pharmacological treatment) 5 (CRC surgically treated)	- Surgery affected the structure of the gut microbiota as demonstrated by multivariate analysis based on Bray-Curtis similarity, and decreased biodiversity - Bacteroidetes was the most abundant phylum in healthy controls and CRC patients before and after chemotherapy - *Fusobacterium*, *Oscillospira*, and *Prevotella* were detected in CRC patients before and after chemotherapy - *Veillonella dispar*, *Prevotella copri,* and *Bacteroides plebeius* were only enriched in CRC patients treated with chemotherapy - Proteobacteria phylum was found in high abundance in CRC patients after surgery
Youssef *et al.*^[43]^	Stomach, pancreas, small intestine, colon, and rectum cancer	Chemotherapy and/or radiotherapy	20 (treated patients) 43 (non-treated patients)	- *Lactobacillaceae* and *Lactobacillus* were observed at higher relative abundances in the treated group compared to the non-treated group
Stringer *et al.*^[44]^	Various types of cancer (colorectal, breast, laryngeal, esophageal, and melanoma)	Chemotherapy (capecitabine, cisplatin/5-FU, FOLFOX4, FOLFOX6, FOLFIRI, 5-FU/folinic acid, paclitaxel, carboplatin and gemcitabine)	16	- Reduced proportions of *Lactobacillus* spp., *Bacteroides* spp., *Bifidobacterium* spp., and *Enterococcus* spp., and increased proportions of *Staphylococcus* spp. and *Escherichia coli* were observed in patients undergoing chemotherapy compared to healthy controls
Tong *et al.*^[45]^	Ovarian cancer	Surgery and chemotherapy (carboplatin, paclitaxel, cisplatin)	18	- The proportions of Bacteroidetes and Firmicutes increased after treatment, while those of Proteobacteria decreased - Anaerobic bacteria, such as *Bacteroides*, *Collinsella,* and *Blautia*, exhibited a significant increase after chemotherapy
Galloway-Peña *et al.*^[47]^	Acute myeloid leukemia	Induction chemotherapy	34	- Loss of bacterial diversity during chemotherapy - Decreased bacterial diversity at baseline was associated with a higher risk of infection - Chemotherapy treatment led to increased abundances of the genus *Lactobacillus* - The gut microbiota of patients treated with chemotherapy was dominated by a single taxon, most frequently by opportunistic pathogens (e.g., *Staphylococcus*, *Enterobacter*, and *Escherichia*)
Han *et al.*^[51]^	Acute myelogenous leukemia, acute lymphoblastic leukemia, myelodysplastic syndrome	Myeloablative regimens (busulfan + cyclophosphamide and total body irradiation + cyclophosphamide) then sequential intensified regimen (fludarabine + cytarabine + total body irradiation + cyclophosphamide + etoposide)	141	- Proteobacteria, Gammaproteobacteria, Enterobacteriales*,* and *Enterobacteriaceae* were associated with aGvHD - Lower microbiota diversity in the aGvHD group compared with the non-aGvHD group - The gut microbiota and conditioning might induce aGvHD by influencing the T regulatory/T helper 17 cell balance
Montassier *et al.*^[52]^	Non-Hodgkin’s lymphoma	Myeloablative conditioning regimen (high dose carmustine, etoposide, aracytine, and melphalan)	28	- Chemotherapy-related decrease in Firmicutes and Actinobacteria and increase in Proteobacteria
Montassier *et al.*^[53]^	Non-Hodgkin’s lymphoma	Myeloablative conditioning regimen (high dose carmustine, etoposide, aracytine, and melphalan)	8	- Bacterial diversity decreased after chemotherapy - Drastic decrease in Firmicutes (in particular, *Faecalibacterium*, *Blautia,* and *Roseburia*) and *Bifidobacterium* after treatment - The relative abundance of *Bacteroides* increased during chemotherapy, as well as that of Proteobacteria - A shift from Gram-positive to Gram-negative bacteria was observed
Rashidi *et al.*^[54]^	Acute myeloid leukemia	Chemotherapy	52	- Higher relative abundances of *Bacteroides* and lower amounts of *Faecalibacterium* and *Alistipes* were detected up to 6 months after chemotherapy
Rajagopala *et al.*^[55]^	Acute lymphoblastic leukemia in pediatric patients	Chemotherapy	32	- Microbiota diversity and richness were significantly lower at diagnosis and during chemotherapy in comparison with healthy controls - The abundance of mucolytic gram-positive anaerobic bacteria, including *Ruminococcus gnavus* and *Ruminococcus torques*, tended to increase during the chemotherapy regimen - At diagnosis, higher proportions of Bacteroidetes (particularly *Bacteroides*) and lower proportions of *Faecalibacterium* were found in patients compared with healthy controls - *Alistipes* proportions decreased substantially during chemotherapy, while *Lachnospiraceae* increased during treatment

aGvHD: Acute graft *vs.* host disease; CID: chemotherapy-induced diarrhea; CRC: colon-rectal cancer; FOLFIRI: folinic acid + fluorouracil + irinotecan; FOLFOX: folinic acid + fluorouracil + oxaliplatin; FU: fluorouracil; HSCT: hematopoietic stem cell transplantation.

In parallel, some research has focused on the GM recovery processes in adult and pediatric patients affected by hematologic malignancies undergoing hematopoietic stem cell transplantations (HSCT). HSCT can lead to several life-threatening complications, such as graft-versus-host disease (GvHD, *i.e.*, when alloreactive donor T cells attack host organs, such as skin, liver, and gut), and local and systemic infections. In this context, several studies showed that treatment-related GM unbalances are associated with poor clinical outcomes^[48]^. Indeed, HSCT practices significantly affect GM homeostasis with a reduction in the diversity and sometimes monodominance by Proteobacteria, *Enterococcus,* or *Streptococcus*^[31,32,49-53]^. Notably, chemotherapy treatments in adult patients have been found to trigger a lasting shift in the GM, with higher relative abundances of *Bacteroides* and lower proportions of *Faecalibacterium* and *Alistipes* detected up to 6 months of follow-up^[54]^. Similar results were confirmed in pediatric patients with various hematological malignancies who underwent HSCT^[31,32]^. Their GM profile was analyzed before and up to 4 months after HSCT, showing the presence of severe dysbiosis, as well as the invasion of newly acquired bacterial species. According to the authors, only 10% of pre-existing species resisted after HSCT, with *Bacteroides* spp. being the most represented among the persistent ones. Also, a decrease in the relative abundance of health-associated taxa, such as *Faecalibacterium* and *Ruminococcus*, was found after HSCT. In general, patients who did recover a healthy GM configuration after HSCT showed a better prognosis, while non-recoverers showed an increased risk of infections, aGvHD, and relapse^[24,32]^. Conflicting results have also been reported regarding the timing of GM recovery, *i.e.*, return to a layout similar to the pre-treatment one. Some studies reported that total bacterial abundance was restored in a few days^[40]^, while in others, a more persistent shift was found, and the GM recovered its initial richness and metabolic capability several months after treatment^[31,54,55]^. These differences in the speed and extent of recovery could be explained by GM layouts before treatment. For example, studies carried out in different contexts have consistently shown that a high-diversity GM is more stable and resilient to perturbations^[56-58]^.

Again, *Bacteroides* was identified as a key player, potentially capable of fostering the re-establishment of the microbial community. In fact, it was preserved during anticancer treatments, resisting not only the perturbations of chemotherapy but also those of antimicrobial therapy. Regarding this last point, a brilliant study by Chng *et al.*^[19]^ found 21 bacterial species with robust associations with post-antibiotic therapy recovery, in particular belonging to the *Bacteroides* genus - *i.e.*, *B. uniformis*, *B. thetaiotaomicron*, *B. stercoris*, *B. egghertii*, *B. coprocola*, *B. caccae*, and *B. intestinalis*. The reason for the persistence of *Bacteroides* during and after treatments may lie in its ability to penetrate the colonic mucus layer and reside within the crypt channels, a region that is more protected and less susceptible to stressors^[59,60]^. Not surprisingly, *Bacteroides fragilis* mutants for carbohydrate utilization systems that are unable to colonize the mucus layer are also less resistant to intestinal perturbations, such as antibiotic treatments and pathogen infections^[60]^. As suggested elsewhere, the breakdown of mucins and complex polysaccharides^[61]^ could be one of the functions that allow members of the Bacteroidetes phylum to stabilize the GM community^[62]^, thus acting as “primary gut species” after perturbations, which contribute to microbiota repopulation^[19,63]^.

## INTERVENTION STRATEGIES TO PROMOTE THE RECOVERY OF GM AFTER CHEMOTHERAPY

Nowadays, GM has effectively become a target of clinical practice in cancer management^[64,65]^. Its close relationship with host well-being has paved the way for the development of precision personalized intervention strategies aimed at promoting more resilient healthy GM configurations associated with a better prognosis^[22,66,67]^. Here, we briefly discuss the potential of prebiotics, probiotics, and fecal microbiota transplantation (FMT), as GM manipulation tools to promote its recovery after chemotherapy treatment^[66,68,69]^.

Prebiotics are defined as “a substrate that is selectively utilized by host microorganisms conferring a health benefit”^[70]^. Most of the prebiotics currently used are based on carbohydrates such as inulin, fructo-oligosaccharides, galacto-oligosaccharides, lactulose, and human milk oligosaccharides^[68,71-73]^. However, other substances, such as polyphenols^[74]^ and polyunsaturated fatty acids^[75]^, are being studied for their beneficial effects on host health. These compounds pass through digestion in the small intestine, reaching the colon virtually unaffected, where they can be fermented by numerous bacterial taxa into SCFAs^[76]^. Although the information on the use of prebiotics in cancer patients is currently limited, they undoubtedly represent a means of promoting GM resistance and resilience^[77]^. In particular, their metabolism is known to involve the establishment of syntrophic cross-feeding interactions^[77,78,79]^, which are essential for the ecological health of GM, and could therefore favor the persistence and/or repopulation of beneficial commensals for more rapid restoration of microbial diversity and abundance.

Probiotics are “live microorganisms that, when administered in adequate amounts, confer a health benefit on the host”^[80]^. Probiotic intake can restore the GM composition and its health-associated functions, limiting pathogens or unhealthy microbial expansions^[81,82]^. The underlying mechanisms include competition for receptor and binding sites, promotion of intestinal mucosa integrity, and production of a range of molecules, including antimicrobial agents, to name a few^[82-84]^. Again, there is little information on the potential of probiotics to specifically promote GM recovery after chemotherapy, but several trials have explored their efficacy in improving clinical outcomes^[21,65]^. Notably, a recent randomized, double-blind, placebo-controlled trial reported that oral administration of a mixture of six viable probiotic strains of lactobacilli and bifidobacteria reduced levels of pro-inflammatory cytokines (*i.e.*, TNF-α, IL-17A, IL-17C, IL-22, and IL-12), but also of IL-10, and prevented post-surgical complications in patients with colorectal cancer (NCT03782428)^[85]^. It should be noted that, although generally considered an anti-inflammatory cytokine, IL-10 has been shown to play a dual role in immunology, as well as tumor pathogenesis and/or progression, with increased levels associated with colorectal cancer progression and poor patient survival^[86-88]^. In addition, a phase II randomized clinical trial showed that an oral probiotic cocktail (containing *Lactobacillus plantarum* MH-301, *Bifidobacterium animalis* subsp. *lactis* LPL-RH, *Lactobacillus rhamnosus* LGG-18, and *Lactobacillus acidophilus*) could alleviate the severity of oral mucositis in patients with nasopharyngeal cancer treated with radiotherapy and chemotherapy by regulating GM dysbiosis and enhancing immune system response (NCT03112837)^[89]^. However, as discussed above, the role of probiotics, especially *Bifidobacterium* spp., may not be entirely favorable during chemotherapy treatments, making the conduct of further clinical studies extremely important. In particular, future studies should investigate the effects of the early intake of probiotics as “GM pre-conditioning” on chemotherapy outcomes and the occurrence of side effects. Administration of the traditional probiotic *E. coli* Nissle 1917 could also be a promising approach for colorectal cancer control, possibly due to its pro-apoptotic effect through upregulation of PTEN (phosphatase and tensin homolog) and Bax and downregulation of AKT1^[90]^. However, with specific regard to GM resilience, it should be noted that the choice of probiotics to be administered should be rationally guided by the knowledge of which are the keystone species associated with GM recovery, which most likely do not include lactobacilli and bifidobacteria (generally subdominant taxa if not absent in adult GMs) but the so-called next-generation probiotics or live biotherapeutics^[91]^. For example, some *Bacteroides* species, such as *B. fragilis* and *B. thetaiotaomicron,* have shown intriguing therapeutic effects on immune derangement and intestinal epithelial barrier impairment, possibly favoring a healthy repopulation of the gut^[19,92-95]^. Additionally, *Bacteroides xylanisolvens* DSM 23964 has been tested in a phase I clinical trial. Heat-inactivated preparations of this organism are hypothesized to improve therapeutic response and cancer immune surveillance by increasing Thomsen-Friedenreich α-specific IgM^[96]^, but the impact on GM recovery is currently unknown.

FMT consists of the transfer of healthy donor stools into the gastrointestinal tract of a patient to improve the dysbiotic state by increasing the overall diversity and restoring the functionality of the GM^[97]^. FMT is currently used for the treatment of recurrent *C. difficile* infection^[98]^ when antibiotics (e.g., vancomycin) and monoclonal antibodies (e.g., bezlotoxumab) fail, as suggested by international guidelines^[99,100]^. In this context, its efficacy rate is between 80% and 90%, as reported by several meta-analyses and randomized clinical trials^[101-104]^. However, some concerns regarding the long-term safety of FMT are emerging, particularly the risk of transfer of pathogens and antibiotic-resistant genes from donor to recipient and/or the occurrence of autoimmunological disorders, which makes the choice of an appropriate donor of utmost importance^[105,106]^. Furthermore, another important issue concerns the viability of anaerobic microbes, of which GM is largely composed. For example, Papanicolas *et al.*^[107]^ found that the practice of preparing material for FMT in ambient air profoundly affected the microbial viability, disproportionally reducing the abundance of anaerobic commensals (including the health-associated taxa *F. prausnitzii* and *Eubacterium hallii*) and the biosynthetic capacity of important anti-inflammatory metabolites. As regards anticancer chemotherapy, as expected, exhaustive information on the application of FMT is not yet available, but some clinical trials have been completed and others are still ongoing. For example, in the single-arm phase II multicenter study by Malard *et al.* (NCT02928523)^[108]^, 25 patients with acute myeloid leukemia were successfully treated with autologous FMT to restore GM dysbiosis and increase biodiversity. In particular, FMT facilitated the restoration of high proportions of health-associated taxa, such as *Lachnospiraceae*, *Ruminococcaceae,* and other Clostridiales (generally dominant in the adult GM), while the decrease of pro-inflammatory taxa belonging to the *Enterobacteriaceae* and *Enterococcaceae* families, which instead predominated during chemotherapy potentially undermining GM recovery.

## CONCLUSION

In the present review, we discussed the available literature on GM dynamics during anticancer chemotherapy, one of the most detrimental stressors to which the human body and its microbial counterpart can be exposed. While the devastating impact of chemotherapy on GM is well established, especially in terms of biodiversity reduction and loss of health-associated taxa, with potential expansion of pathobionts, the theme of GM resilience and recovery has not yet been sufficiently explored. Indeed, most of the available evidence concerns the ability of only one GM genus, *Bacteroides*, to withstand environmental stresses and help rebuild the microbial community. Although GM dynamics in a context such as cancer may seem at first glance to be of little relevance, identifying taxa associated with ecological recovery and understanding their interactions for a rapid, complete, and healthy community restocking would be of paramount importance as it could guide the rational design of microbiome-based adjuvant strategies to promote response to therapy and limit long-term negative consequences for oncological patients’ health. In this regard, GM manipulation tools such as prebiotics, probiotics, and FMT have shown promising results, but again, no particular attention has been paid to whether and to what extent and how quickly they allow the recovery of a eubiotic GM. Future studies should unravel such aspects for a revolution in the clinical approach, which places the evidence and the mechanisms of action as the basis of the choice of intervention strategies.

## DECLARATIONS

### Copyright

© The Author(s) 2023.
